# Supplementary material for: Development and validation of a nomogram for predicting cefoperazone/sulbactam-induced hypoprothrombinaemia in Hospitalized adult patients
Source: PLoS One. 2023 Sep 21;18(9):e0291658. doi: 10.1371/journal.pone.0291658 (PMC10513251; doi:10.1371/journal.pone.0291658)
Supplement: S3 File — (PDF) [file pone.0291658.s003.pdf]

R RECORD-0829

```
> ###################################################################################################################################################################################################
>
> ##列线图（Alignment Diagram），又称诺莫图（Nomogram图）,可将Logistic回归或Cox回归的结果进行可视化呈现。
> ##COX回归模型，又称“比例风险回归模型(proportional hazards model，简称Cox模型)”，是由英国统计学家D.R.Cox(1972)年提出的一种半参数回归模型。
> ## C-index ，即一致性指数（concordance index），用来评价模型的预测能力。c指数是指所有病人对子中预测结果与实际结果一致的对子所占的比例。
>
> ###################################################################################################################################################################################################
>
>
>
> #安装需要的包 第一次执行一下就行
> # install.packages("rms")
> # install.packages("pROC")
> # install.packages("ggplot2")
> # install.packages("ResourceSelection")
>
>
> #引入需求的包
> library(rms)                    #回归模型包，包含Nomogram函数
> library(pROC)                   #显示和分析 ROC 曲线
> library(ggplot2)
> library(ResourceSelection)
>
>
> # 训练数据从 xxx.csv中读取 header = T 表示包含表头 sep = "," 表示用 , 分割
> data_train <-
+   read.csv("train.csv",
+             header = T,
+             sep = ",")
> data_test <-
+   read.csv("test.csv",
```

```
+             header = T,
+             sep = ",")
>
> # NameA ~ NameB + NameC + NameD 定义了一个公式 A = kB + mC +nD
> # 其中(k,m,n)都是要拟合的参数
> # NameA NameB NameC NameD 表示csv里面的列名 cvs中的列名中不能有空格
>
>
> correlation_fun <-
+   Coagulation_disorders_or_bleeding ~ Cumulative_DDDs + Surgery + Baseline_PLT + Baseline_liver_function + NRS2002
>
> # 通过上面公式 定义一下逻辑回归模型 sigmoid(fun) 并拟合参数
>
> glm_model <-
+   glm(
+     correlation_fun,
+     data = data_train,
+     family = binomial(link = "logit"),
+     x = T
+   )
>
> # 简单打印一下拟合后的模型
> summary(glm_model)

Call:
glm(formula = correlation_fun, family = binomial(link = "logit"),
    data = data_train, x = T)

Coefficients:
                        Estimate Std. Error z value Pr(>|z|)
(Intercept)             -6.30171    0.64532  -9.765  < 2e-16 ***
Cumulative_DDDs          0.14986    0.02544   5.891 3.83e-09 ***
Surgery                  1.66372    0.36187   4.598 4.27e-06 ***
Baseline_PLT             0.91298    0.41256   2.213 0.026900 *
Baseline_liver_function  2.51464    0.67741   3.712 0.000206 ***
NRS2002                  2.83160    0.42778   6.619 3.61e-11 ***
---
Signif. codes:  0 ‘***’ 0.001 ‘**’ 0.01 ‘*’ 0.05 ‘.’ 0.1 ‘ ’ 1

(Dispersion parameter for binomial family taken to be 1)

```

```
    Null deviance: 371.07  on 426  degrees of freedom
Residual deviance: 222.68  on 421  degrees of freedom
AIC: 234.68

Number of Fisher Scoring iterations: 6

>
> # 训练集P值
> pvalue_train <- hoslem.test(glm_model$y, fitted(glm_model), g=4)
> pvalue_train

    Hosmer and Lemeshow goodness of fit (GOF) test

data:  glm_model$y, fitted(glm_model)
X-squared = 1.4903, df = 2, p-value = 0.4747

>
> glm_model_test <-
+   glm(
+     correlation_fun,
+     data = data_test,
+     family = binomial(link = "logit"),
+     x = T
+   )
>
> # 测试集P值
> pvalue_test <- hoslem.test(glm_model_test$y, fitted(glm_model_test), g=4)
> pvalue_test

    Hosmer and Lemeshow goodness of fit (GOF) test

data:  glm_model_test$y, fitted(glm_model_test)
X-squared = 1.9124, df = 2, p-value = 0.3844

>
>
> #下面我们用来用拟合好的模型算一下 c_index
> # 注意一下 predic这个函数 第一个参数是glm_model:用来评估的模型 第二个参数 newdata = data_train表示用来测试的数据 即 Coagulation_disorders_or_bleeding = glm_model(data_train) type=response, 表示输出结果预测响应变量为1的概率
> # 再说一下 rcorrcens这个函数 第一个是一个公式，第二个是公式里面的变量所来的数据 这个公式是: 真实的Coagulation_disorders_or_bleeding ~ 预测的真实的Coagulation_di
```

```
sorders_or_bleeding
>
> #先计算训练集的
> c_index_train <-
+   rcorrcens(
+     Coagulation_disorders_or_bleeding ~ predict(glm_model, newdata = data_train, type = "response"),
+     data = data_train
+   )
>
> #再计算测试集的
> c_index_test <-
+   rcorrcens(
+     Coagulation_disorders_or_bleeding ~ predict(glm_model, newdata = data_test, type = "response"),
+     data = data_test
+   )
>
> print(c_index_train)

Somers' Rank Correlation for Censored Data    Response variable:Coagulation_disorders_or_bleeding

                                                                  C   Dxy  aDxy
predict(glm_model, newdata = data_train, type = "response") 0.909 0.818 0.818
                                                                 SD     Z P   n
predict(glm_model, newdata = data_train, type = "response") 0.035 23.64 0 427
> print(c_index_test)

Somers' Rank Correlation for Censored Data    Response variable:Coagulation_disorders_or_bleeding

                                                                 C   Dxy  aDxy
predict(glm_model, newdata = data_test, type = "response") 0.837 0.674 0.674
                                                                SD    Z P   n
predict(glm_model, newdata = data_test, type = "response") 0.101 6.68 0 221
>
>
>
> #下面我们用来用拟合好的模型算画下 ROC曲线
>
> #训练集的ROC曲线
```

```
>
> roc_train <-
+   roc(
+     Coagulation_disorders_or_bleeding ~ predict(glm_model, newdata = data_train, type = "response"),
+     data = data_train
+   )
Setting levels: control = 0, case = 1
Setting direction: controls < cases
>
> plot(
+   roc_train,
+   print.auc = TRUE,
+   #输出AUC值
+   print.thres = TRUE,
+   #输出cut-off值
+   main = "ROC CURVE TRAINING",
+   #设置图形的标题
+   col = "red",
+   #曲线颜色
+   print.thres.col = "black",
+   #cut-off值字体的颜色
+   identity.col = "blue",
+   #对角线颜色
+   identity.lty = 1,
+   identity.lwd = 1
+ )
>
>
> #训练集roc的详细结果
> roc_train_result <- coords(roc_train,"best" , ret="all" , transpose =  FALSE)
> as.matrix(roc_train_result)
          threshold specificity sensitivity  accuracy  tn tp fn fp       npv
threshold 0.1580495   0.8138889   0.8507463 0.8196721 293 57 10 67 0.9669967
                ppv       fdr       fpr       tpr       tnr       fnr
threshold 0.4596774 0.5403226 0.1861111 0.8507463 0.8138889 0.1492537
          1-specificity 1-sensitivity 1-accuracy     1-npv     1-ppv precision
threshold     0.1861111     0.1492537  0.1803279 0.0330033 0.5403226 0.4596774
             recall   youden closest.topleft
threshold 0.8507463 1.664635      0.05691402
>
> ##训练集的95置信区间
```

```
> ci_train <- ci(auc(roc_train))
>
> print(ci_train)
95% CI: 0.8747-0.9429 (DeLong)
>
> #测试集的ROC曲线
>
> roc_test <-
+   roc(
+     Coagulation_disorders_or_bleeding ~ predict(glm_model, newdata = data_test, type = "response"),
+     data = data_test
+   )
Setting levels: control = 0, case = 1
Setting direction: controls < cases
>
> plot(
+   roc_test,
+   print.auc = TRUE,
+   #输出AUC值
+   print.thres = TRUE,
+   #输出cut-off值
+   main = "ROC CURVE EXTERNAL VALIDATION",
+   #设置图形的标题
+   col = "red",
+   #曲线颜色
+   print.thres.col = "black",
+   #cut-off值字体的颜色
+   identity.col = "blue",
+   #对角线颜色
+   identity.lty = 1,
+   identity.lwd = 1
+ )
>
> #测试集roc的详细结果
> roc_test_result <- coords(roc_test , "best" , ret="all" , transpose =  FALSE)
> as.matrix(roc_test_result)
          threshold specificity sensitivity  accuracy  tn tp fn fp       npv
threshold 0.1813226   0.7889447   0.8181818 0.7918552 157 18  4 42 0.9751553
          ppv fdr       fpr       tpr       tnr       fnr 1-specificity
threshold 0.3 0.7 0.2110553 0.8181818 0.7889447 0.1818182     0.2110553
          1-sensitivity 1-accuracy      1-npv 1-ppv precision    recall
```

```
threshold     0.1818182  0.2081448 0.02484472   0.7       0.3 0.8181818
            youden closest.topleft
threshold 1.607127      0.07760218
>
>
> #测试集的95置信区间
> ci_test <- ci(auc(roc_test))
> print(ci_test)
95% CI: 0.7359-0.9383 (DeLong)
>
>
> #下面来算一下 列线图
>
> #Surgery内容标签更改
> data_train$Surgery <- factor(data_train$Surgery,
+                                   levels = c(0,1),
+                                   labels = c("no","yes")
+                                   )
>
> #Baseline_liver_function内容标签更改
> data_train$Baseline_liver_function <- factor(data_train$Baseline_liver_function,
+                              levels = c(0,1),
+                              labels = c("no","yes")
+ )
>
> #NRS2002内容标签更改
> data_train$NRS2002 <- factor(data_train$NRS2002,
+                              levels = c(0,1),
+                              labels = c("no","yes")
+ )
>
> #Baseline_PLT内容标签更改
> data_train$Baseline_PLT <- factor(data_train$Baseline_PLT,
+                                   levels = c(0,1),
+                                   labels = c("no","yes")
+ )
>
> #Cumulative_DDDs名称标签更改
> attr(data_train[["Cumulative_DDDs"]], "label") <- "Cumulative DDDs"
>
> #Baseline_liver_function名称标签更改
```

```
> attr(data_train[["Baseline_liver_function"]], "label") <- "Baseline hepatic dysfunction"
>
> #Baseline_PLT名称标签更改
> attr(data_train[["Baseline_PLT"]], "label") <- "Baseline PLT count ≤ 50×10^9/L"
>
> #Baseline_PLT名称标签更改
> attr(data_train[["NRS2002"]], "label") <- "Nutritional risk"
>
>
> #整合数据
> ddist <- datadist(data_train)
> options(datadist = 'ddist')
>
> lrm_model <- lrm(correlation_fun, data = data_train,x=T, y=T)
> summary(lrm_model)
             Effects              Response : Coagulation_disorders_or_bleeding

 Factor                           Low High Diff. Effect   S.E.    Lower 0.95
 Cumulative_DDDs                  6   14    8     1.19880 0.20349 0.80001
  Odds Ratio                      6   14    8     3.31630      NA 2.22560
 Surgery - yes:no                 1    2   NA     1.66370 0.36187 0.95447
  Odds Ratio                      1    2   NA     5.27890      NA 2.59730
 Baseline_PLT - yes:no            1    2   NA     0.91298 0.41257 0.10437
  Odds Ratio                      1    2   NA     2.49170      NA 1.11000
 Baseline_liver_function - yes:no 1    2   NA     2.51460 0.67742 1.18690
  Odds Ratio                      1    2   NA    12.36200      NA 3.27700
 NRS2002 - yes:no                 1    2   NA     2.83160 0.42779 1.99310
  Odds Ratio                      1    2   NA    16.97300      NA 7.33850
 Upper 0.95
  1.5977
  4.9416
  2.3730
 10.7290
  1.7216
  5.5935
  3.8424
 46.6350
  3.6701
 39.2540
```

```
> # plot nomogram
> nom <-
+   nomogram(
+     lrm_model,
+     fun = plogis,
+     fun.at = c(.001, .01, .05, 0.158, seq(.3, .9, by = .2), .95, .99, .999),
+     lp = F,
+     funlabel = "Risk of hypoprothrombinaemia"
+   )
> # 如果想直接画到一个pdf里面 可以打开这句
> # pdf(file = "nomogram.pdf",width = 8, height = 6)
> plot(nom,xfrac=.45)
>
>
> #训练集的校正曲线
>
> cal_train <- calibrate(lrm_model,method='boot',B=1000)
> plot(cal_train,
+      xlim = c(0,1),
+      ylim = c(0,1),
+      xlab = "Prediced Probability",
+      ylab = "Observed Probability",
+      cex.lab=1.2, cex.axis=1, cex.main=1.2, cex.sub=0.8,
+      #subtitles = FALSE,
+      legend = FALSE
+ )

n=427   Mean absolute error=0.01   Mean squared error=0.00026
0.9 Quantile of absolute error=0.021

> lines(cal_train[,c("predy","calibrated.corrected")],
+       type = 'l', #连线的类型，可以是"p","b","o"
+       lwd = 3, #连线的粗细
+       pch = 16, #点的形状，可以是0-20
+       col = "#2166AC") #连线的颜色
> lines(cal_train[,c("predy","calibrated.orig")],type="l",pch=16,lwd=3,col="tomato")
> abline(0,1,
+        lty = 2, #对角线为虚线
+        lwd = 2, #对角线的粗细
+        col = "#224444") #对角线的颜色
> legend(0.65,0.3,
```

```
+         c("Apparent","Bias-corrected","Ideal"),
+         lty = c(2,1,1),
+         lwd = c(2,3,3),
+         col = c("black","#2166AC","tomato"),
+         bty = "n"
+ )
>
> title(main = "TRAINING COHORT")
>
>
> #测试集的校正曲线
>
> lrm_model <- lrm(correlation_fun, data = data_test,x=T, y=T)
> cal_test <- calibrate(lrm_model,method='boot',B=1000)
> plot(cal_test,
+       xlim = c(0,1),
+       ylim = c(0,1),
+       xlab = "Prediced Probability",
+       ylab = "Observed Probability",
+       cex.lab=1.2, cex.axis=1, cex.main=1.2, cex.sub=0.8,
+       #subtitles = FALSE,
+       legend = FALSE
+ )

n=221   Mean absolute error=0.016   Mean squared error=0.00038
0.9 Quantile of absolute error=0.032

> lines(cal_test[,c("predy","calibrated.corrected")],
+        type = 'l', #连线的类型, 可以是"p","b","o"
+        lwd = 3, #连线的粗细
+        pch = 16, #点的形状, 可以是0-20
+        col = "#2166AC") #连线的颜色
> lines(cal_test[,c("predy","calibrated.orig")],type="l",pch=16,lwd=3,col="tomato")
> abline(0,1,
+         lty = 2, #对角线为虚线
+         lwd = 2, #对角线的粗细
+         col = "#224444") #对角线的颜色
> legend(0.65,0.3,
+         c("Apparent","Bias-corrected","Ideal"),
+         lty = c(2,1,1),
+         lwd = c(2,3,3),
```

```
+         col = c("black","#2166AC","tomato"),
+         bty = "n"
+ )
>
> title(main = "EXTERNAL VALIDATION COHORT")
```
