# Supplementary material for: Development and validation of a nomogram for predicting cefoperazone/sulbactam-induced hypoprothrombinaemia in Hospitalized adult patients
Source: PLoS One. 2023 Sep 21;18(9):e0291658. doi: 10.1371/journal.pone.0291658 (PMC10513251; doi:10.1371/journal.pone.0291658)
Supplement: Attachment — Submitted filename: Response to Reviewers.docx [file pone.0291658.s004.docx]

Response to Reviewers

**Academic Editor**

Response1: Our manuscript has been modified according to PLOS ONE style templates.

Response2: If the funders had no role, as stated in the manuscript: "The funders had no role in study design, data collection and analysis, decision to publish, or preparation of the manuscript."

Response3: We have changed the data availability statement in the cover letter.

Response4: The corresponding author's ORCID iD is https://orcid.org/0009-0001-9586-3719.

Response5: The ethics statement have been amended ethics statement ethics statement in the Methods section of the manuscript.

Response6: It has been confirmed that only Tables 1 and 2 are referred to for the manuscript.

Response7: Supporting information files have been added at the end of the manuscript.

Response8: Papers that have been retracted are not cited in the manuscript. According to the suggestions of reviewer #1, references [7], [31] and [34] are deleted, references [6], [11], [20], [21] and [25] are replaced, and references [29] are added in the manuscript.

**Reviewer** **#1**

Response1: The nomogram model was externally verified in the Third Affiliated Hospital of Xi 'an Medical University, with an AUC of 0.837 (95%CI=0.736–0.938), indicating that the predictive ability of the nomogram for cefoperazone/sulbactam-induced hypoprothrombinaemia in other centres is equally robust. The manuscript has been modified accordingly.

Response2: The HIS can sort out and integrate the data information from laboratory information system (LIS), picture archiving and communication system (PACS) and radiology information system (RIS) to avoid inaccuracies or missing data and ensure data reliability.

Response3: We have updated the references and removed the old ones. References [7], [31] and [34] were deleted from the original manuscript, references [6], [11], [20], [21] and [25] were replaced, and references [29] were added.

**Reviewer #2**

**Study design and collection**

This study is retrospective and patient informed consent has been waived in ethical statement. We have amended our statements regarding patient informed consent.

The Naranjo ADR Probability Scale was developed to help standardize assessment of causality for all adverse drug reactions. The Naranjo Scale contains 10 subscales, with Yes, No, and Not known or not done options for each item. Among them, the "No" option of the 2nd and 4th subscales has a score of -1, and the "Yes" option of the 5th and 6th subscales has a score of -1, and there may be cases where the score is less than or equal to 0. View the Naranjo scale of use at <https://www.evidencio.com/models/show/661.>

**Statistical Analysis**

Before conducting the analysis, it was confirmed that the assumed preconditions for logistic regression had been met.

**Baseline Characteristics of the Patients**

Among 893 patients, 378 warning signs were extracted through the CHPS and 283 patients with no causal relationship with cefoperazone/sulbactam were excluded after independent review by two clinical pharmacists using the Naranjo Scale. Because the 283 patients with warning signs could be related to factors other than cefoperazone/sulbactam, they were not included in the final analysis. Finally, 610 patients were included in the analysis.

Among the 378 patients with warning signs, 283 patients were excluded from possible factors other than cefoperazone/sulbactam, and 95 patients were finally determined to have cefoperazone/sulbactam-induced hypoprothrombinaemia (**Fig 1**). This study eventually included 610 patients in the analysis, who were randomized at a 7:3 ratio into the training (n=427) and validation (n=183) groups. The training group (n=427) conducted logistic regression analysis and screened independent predictive factors to establish nomogram model.

**Discussion**

The discussion has been partially moved to the introduction. The discussion has been partially moved to the introduction. The sentence “In our study, the overall incidence rate of cefoperazone/sulbactam-induced hypoprothrombinaemia among inpatients was 10.64%(95/893), which was consistent with that in the literature.” was also deleted.

The last subtitle has been corrected to conclusion.
